# A Role for Host Activation-Induced Cytidine Deaminase in Innate Immune Defense against KSHV

**DOI:** 10.1371/journal.ppat.1003748

**Published:** 2013-11-07

**Authors:** Elena Bekerman, Diana Jeon, Michele Ardolino, Laurent Coscoy

**Affiliations:** Division of Immunology and Pathogenesis, Department of Molecular and Cell Biology, University of California, Berkeley, Berkeley, California, United States of America; University of Southern California Keck School of Medicine, United States of America

## Abstract

Activation-induced cytidine deaminase (AID) is specifically induced in germinal center B cells to carry out somatic hypermutation and class-switch recombination, two processes responsible for antibody diversification. Because of its mutagenic potential, AID expression and activity are tightly regulated to minimize unwanted DNA damage. Surprisingly, AID expression has been observed ectopically during pathogenic infections. However, the function of AID outside of the germinal centers remains largely uncharacterized. In this study, we demonstrate that infection of human primary naïve B cells with Kaposi's sarcoma-associated herpesvirus (KSHV) rapidly induces AID expression in a cell intrinsic manner. We find that infected cells are marked for elimination by Natural Killer cells through upregulation of NKG2D ligands via the DNA damage pathway, a pathway triggered by AID. Moreover, without having a measurable effect on KSHV latency, AID impinges directly on the viral fitness by inhibiting lytic reactivation and reducing infectivity of KSHV virions. Importantly, we uncover two KSHV-encoded microRNAs that directly regulate AID abundance, further reinforcing the role for AID in the antiviral response. Together our findings reveal additional functions for AID in innate immune defense against KSHV with implications for a broader involvement in innate immunity to other pathogens.

## Author Summary

Immune responses to pathogens rely heavily on the ability of B cells to generate a unique set of antibodies that can bind and eliminate the pathogen. Activation-induced cytidine deaminase (AID) is the enzyme specifically upregulated in activated B cells to diversify the antibody repertoire by introducing mutations within the antibody coding genes. Curiously, AID expression has been observed outside of activated B cells upon infection with a number of viral and bacterial pathogens. However, in such cases AID function is poorly characterized and often deemed inappropriate since its mutagenic activity can put the cell at risk for oncogenic transformation. In this study, we investigate the expression of AID in response to infection with an oncogenic human pathogen Kaposi's sarcoma-associated herpesvirus (KSHV) and the antibody-independent immune defense it exerts. We show that AID marks infected cells for elimination by natural killer (NK) cells and directly impinges on viral fitness. Furthermore, we uncover novel viral immune evasion strategies employed by KSHV to counteract AID. Together, our findings demonstrate a protective role for AID in the response to infection with an oncogenic virus such as KSHV and suggest that AID may actually limit transformation rather than serve as its culprit.

## Introduction

Herpesviruses have co-evolved with their hosts for millions of years, acquiring means to evade and manipulate host immune responses [1]. Evolutionary success of these viruses is highlighted by their life-long persistence, high prevalence and minimal pathological burden in immunocompetent hosts. In cases of immune suppression, however, these viruses can cause severe disease. Kaposi's Sarcoma Associated Herpesvirus (KSHV) is a member of the human γ-herpesvirus family characterized by lymphotropism and strict host specificity. It is the causative agent of Kaposi's Sarcoma, the most common form of malignancy in AIDS patients, and two lymphoproliferative disorders, primary effusion lymphoma (PEL) and multicentric Castleman's disease (MCD) [2]–[4].

Although KSHV can infect a variety of cell types, B cells serve as the primary reservoir of the virus *in vivo* [5], [6]. KSHV favors establishment of latency upon infection, but may reactivate to undergo lytic replication. During latency viral genome is maintained as a multicopy chromatinized episome tethered to host DNA and no progeny virions are generated. Latent gene expression is restricted to just four protein-coding genes [7], [8] and a cluster of twelve miRNAs [9]–[12], all of which contribute towards promoting cell survival, segregating viral episome during mitosis and suppressing host immune responses. Upon entry into the lytic life cycle, virtually all viral genes (87 have been identified thus far) are activated in a temporal fashion, the viral genome is replicated and progeny virions are produced, generally resulting in death of the infected cell.

Multiple arms of host immunity mount responses against KSHV and are counteracted by numerous lines of viral defense. In particular, the humoral response is instrumental in controlling KSHV through the action of neutralizing antibodies and antibody-dependent cell mediated cytotoxicity [13]–[17]. Expression of activation-induced cytidine deaminase (AID) in germinal center (GC) B cells is crucial for generating high affinity antibodies during the adaptive immune response. This enzyme functions to deaminate cytidine residues within immunoglobulin genes. This activity is required for class-switch recombination (CSR) and somatic hypermutation (SHM), which together contribute to the diversification of the antibody repertoire as well as increase antibody affinity [18]. The rate of mutation within the variable region of immunoglobulin genes during SHM is roughly six orders of magnitude greater than the rate of basal somatic mutation [19]. While AID is preferentially targeted to immunoglobulin loci, other regions of the genome are also susceptible to deamination, albeit to lower degree [20]. Accordingly, AID-dependent mutations and chromosomal translocations within proto-oncogenes such as BCL-6, FAS and MYC contribute directly to malignant transformations [21]–[23].

AID belongs to a larger family of tissue restricted vertebrate RNA/DNA editing enzymes, which include APOBEC1 through APOBEC4 [24]. APOBEC3 genes confer innate immunity to a wide range of retroviruses and help prevent transposition of endogenous transposable elements capable of disrupting host genome integrity [25]. More recently, AID has also been implicated in similar defenses for its ability to restrict retrotransposons as well as the transforming retrovirus Ab-MLV in a mouse model [26]. In parallel with these findings, a growing body of literature documents AID induction outside of GCs in response to a variety of pathogens, further supporting the notion that AID may serve dual functions in both adaptive and innate immunity [27]–[30].

In this study, we set out to examine AID expression in the context of KSHV infection, and to establish whether it can negatively impact KSHV fitness as an innate immune defense strategy. Our findings revealed a consistently rapid upregulation of AID within primary human B cells in response to KSHV infection. AID expression continued to rise throughout the course of the infection in our primary cell culture. Additionally, infected cells upregulated surface ligands for the activating receptor NKG2D, expressed by the cytotoxic lymphocytes including natural killer (NK) cells. Consistent with AID's ability to induce DNA damage, we found NKG2D ligand induction to be dependent on the DNA damage response pathway. We also addressed KSHV latency and reactivation potential in AID expressing cells, and found that prolonged exposure to AID significantly inhibits initiation of the lytic replication program of this virus and severely limits the infectivity of its progeny virions. In parallel, we examined the ability of KSHV-encoded miRNAs to thwart AID-mediated immunity. Our data uncovered two KSHV miRNAs, K12-11 and K12-5 capable of interacting with the 3′UTR of AID and translationally repressing it. Together our data elucidate a critical role for AID in innate immune defense against KSHV and also suggest a role for AID in defense against other viral infections.

## Results

### KSHV infection results in upregulation of AID in primary human tonsillar B cells

**Figure 1:**
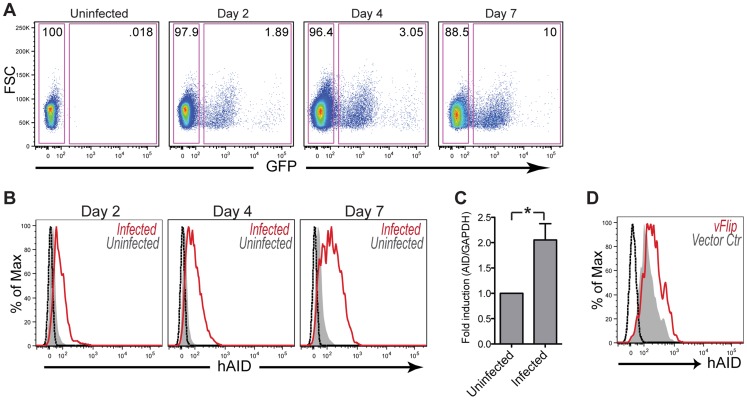
KSHV infection results in upregulation of AID in Primary Human Tonsillar B cells. (**A**) Primary tonsillar cells were infected with KSHV by co-culture with reactivated iSLK.219 for the indicated time. Percent infection of B cells was assessed via flow cytometry by gating on CD19^+^ cells and quantifying infected, GFP^+^ cells (right rectangular gate). (**B**) Intracellular AID expression was assessed in infected, GFP^+^ B cells (red) and uninfected, GFP^−^ B cells (filled gray) using flow cytometry. Black histogram represents unstained control. (**C**) Tonsillar cells were infected by co-culture with reactivated iSLK.219 cells for 2 days. CD19^+^ population was enriched (see figure S1) and subsequently sorted for infected, GFP^+^ and uninfected, GFP^−^ cells. The level of *AID* transcript was measured by qRT-PCR. Shown are mean relative values ± SD from 4 independent patients (*p<.01 by two-tailed, paired student t-test). (**D**) Tonsillar cells were transiently transfected with vFLIP (red) or vector control (gray) plasmids. 48 hrs post transfection CD19^+^, GFP^+^ (transfection marker) cells were analyzed for intracellular AID by flow cytometry. Black histogram represents unstained control.

To assess expression of AID upon KSHV infection of primary B cells, we took advantage of a recently developed co-culture system, where B cells are infected via direct contact with reactivated iSLK.219 cells. These cells harbor latent KSHV marked by constitutive GFP expression and induction of RFP expression upon doxycycline-stimulated lytic reactivation [31]. Co-culture of primary human tonsillar cells with reactivated iSLK.219 cells gave rise to a reproducible population of infected B cells as measured by GFP expression (Figure 1A), with a gradual increase in the fraction of infected cells over time. While KSHV was also capable of infecting tonsillar T cells in our co-culture, T-cell infection has previously been shown to be abortive [32]. We therefore focused our studies on the physiologically relevant CD19^+^ B cell population.

KSHV infection resulted in AID induction in tonsillar B cells as early as on day two of co-culture, as assessed by flow cytometric analysis of GFP-positive versus GFP-negative B cells (Figure 1B). This expression continued to increase through day 7 post-infection, which marks the limit of the tonsillar cell survival in our cytokine-free culture. Importantly, co-culture of tonsillar B cells with KSHV-negative iSLKs failed to produce a population with elevated AID expression (data not shown). This suggested that KSHV infection was directly responsible for inducing AID rather than selectively infecting and expanding cells with already elevated levels of AID. To investigate whether AID upregulation was initiated at the transcript level, infected GFP-positive tonsillar B cells were sorted and AID mRNA levels were determined by qRT-PCR. As seen in Figure 1C, infection resulted in a significant increase of host *AID* transcript as compared to uninfected cells. While we cannot rule out transcript stabilization to explain this result, previous work supports the role for enhancers and silencers as the primary means of AID regulation in activated B cells [33].

AID expression in GC B cells is normally stimulated by synergistic actions of IL-4 and CD40 ligand, which lead to the activation of JAK/STAT and NF-κB pathways, respectively [34], [35]. Given that latent infection of tonsillar B cells, as determined by GFP-positive, RFP-negative signal via flow cytometry, is sufficient to upregulate AID (Figure 1B) we set out to identify whether expression of a particular latent viral gene may be adequate to induce AID expression. KSHV latent protein vFLIP is an established constitutive activator of NF-κB raising the possibility for a role in AID upregulation [36]. To test this hypothesis we transiently transfected primary B cells with vFLIP, and found that relative to vector control vFLIP was able to induce AID expression in the absence of other viral gene products at 48 hrs post transfection (Figure 1D). This result demonstrates that vFLIP alone is sufficient to turn on cellular pathways responsible for AID expression. Together our results indicate that KSHV infection leads to an early transcriptional upregulation of host AID in a cell intrinsic manner, and that this expression is sustained beyond the early phase of infection.

### KSHV infection leads to upregulation of NKG2D ligands via the DNA damage response pathway

**Figure 2:**
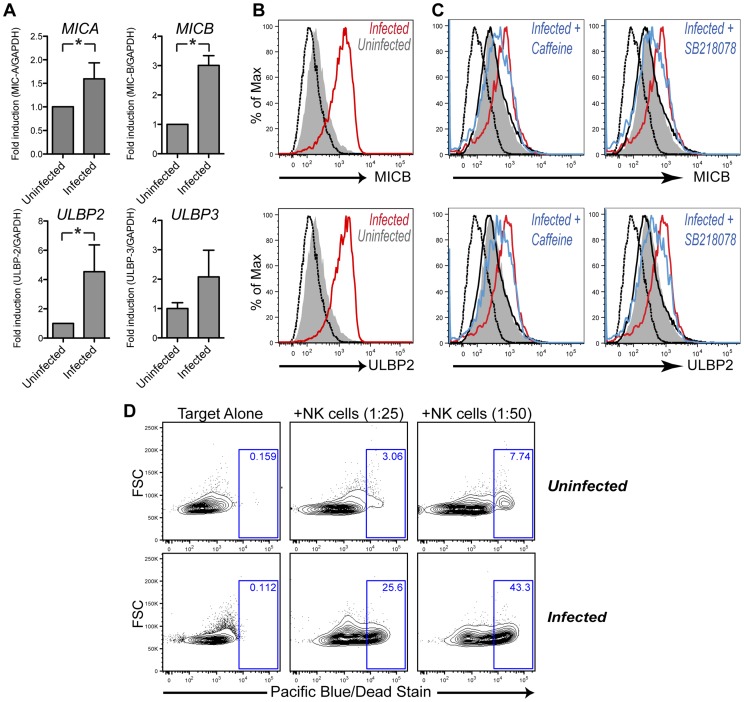
KSHV infection leads to upregulation of NKG2D ligands. (**A**) Tonsillar cells were infected by co-culture with reactivated iSLK.219 cells. CD19^+^ population was enriched and subsequently sorted for infected, GFP^+^ and uninfected, GFP^−^ cells. Relative levels of four detected NKG2D transcripts were measured by qRT-PCR. Shown are mean values ± SD from at least three patients (*p<.01 by two-tailed, paired student t-test). (**B**) CD19^+^ tonsillar B cells were assessed for surface expression of MICB (top panel) and ULBP2 (bottom panel) by flow cytometry. Infected, GFP^+^ cells are depicted in red and uninfected, GFP^−^ cells in filled gray; isotype control is shown in dashed black. (**C**) Infected cells were treated with DNA damage inhibitors, caffeine (middle panel) and SB218078 (right panel) for the duration of the infection. Infected, drug-treated B cells are shown in blue and uninfected drug-treated cells in thin black as compared to vehicle controls depicted same as in (B). (**D**) Tonsillar B cells were sorted for infected, GFP^+^ and uninfected, GFP^−^ cells. GFP^−^, uninfected cells were labeled with CFSE for tracking. Infected or uninfected cells were co-incubated with effector NK-92 cells at specified ratios for 5 hours and assayed for killing by flow cytometry. Percentage of dead target cells is indicated by the blue rectangular gates. All experiments presented in the figure were done on day 4 post infection.

NKG2D ligands are a family of host-encoded proteins that enable NK cells to identify and eliminate damaged or infected cells [37]. It is well established that activation of the DNA damage response pathway leads to the induction of NKG2D ligand expression [38]. Furthermore, previous reports have shown AID and APOBEC3G to activate the DNA damage response upon infection with Ab-MLV and HIV, respectively, resulting in NKG2D ligand induction [26], [39]. AID induction in KSHV infected cells led us to speculate that these cells may also upregulate NKG2D ligands. Of the eight known human NKG2D ligands, we observed increased levels of *MICA*, *MICB*, *ULBP2* and *ULBP3* transcripts by qRT-PCR in the KSHV infected B cell population (Figure 2A). The remaining four ligands either did not differ in their expression or were below the limit of detection in our assay (data not shown). Since NKG2D ligands are known to be regulated post-transcriptionally [40], we wondered whether increased transcript levels correlated with higher expression of ligands at the cell surface. As shown in figure 2B, both ULBP2 and MICB, the two most induced transcripts, were upregulated at the surface of infected B cells. Next, we asked whether up-regulation of NKG2D ligands was due to the activation of the DNA damage response (DDR). Therefore, we treated infected cultures with pharmacological inhibitors of DDR effector molecules such as checkpoint kinase 1 and ataxia telangiectasia mutated kinase. Importantly, both SB218078 (inhibitor of checkpoint kinase 1) and caffeine (inhibitor of ataxia telangiectasia mutated kinase) consistently diminished induction of the analyzed NKG2D ligands within the GFP-positive population, while basal ligand expression in the uninfected cells remained unchanged (Figure 2C).

Since the activating receptor NKG2D has been linked with immune-surveillance of viral infections [41], we wondered whether KSHV-mediated increase of NKG2D ligand expression rendered B cells a better target for NK cell-mediated lysis. Therefore, we sorted infected and uninfected cells as before and co-cultured them with the human NK cell line, NK92. NK92 cells have a high expression of NKG2D and are capable of killing NKG2D ligand-expressing target cells [42], [43]. Using a flow cytometry-based assay we observed an increase in the death of infected compared to uninfected cells (Figure 2D), in accordance with the higher surface expression of NKG2D ligands. Moreover, the extent of observed killing correlated well with the target to effector ratio.

Together, these results demonstrate that KSHV infected primary human B cells, which exhibit elevated AID expression, concurrently upregulate ligands for activating NK cell receptors and become a better target for NK cell recognition and killing. This upregulation is at least partially dependent on the DNA damage response pathway given the diminished ligand induction in presence of DDR inhibitors.

### AID expression causes a defect in lytic reactivation and infectivity but does not affect viral latency

**Figure 3:**
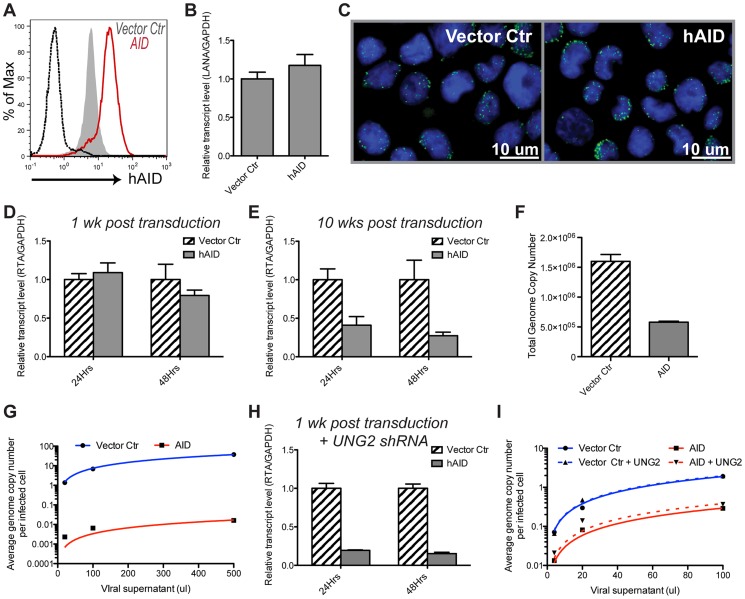
AID expression does not affect viral latency, but results in lytic reactivation and infectivity defects. (**A**) BCBL-1 cell were transduced either with empty vector ctr (filled gray) or AID (red) retroviral constructs, selected for 1 wk until all untransduced cells have died. AID protein expression assessed by flow cytometry. Unstained control is shown in black. See also figure S2A & B. (**B, C**) Empty vector control or AID-expressing BCBL-1 cells were analyzed for *LANA* transcript by qRT-PCR (B) or protein by immunofluorescence staining (C) at 10 wks post selection. Error bars (SD) are derived from triplicates. Shown is one representative experiment out of three performed. LANA staining is depicted in green, DAPI nuclear marker is depicted in blue. (**D, E**) BCBL-1 cells stably transduced with empty vector control or AID for 1 wk (**D**); 10 wks (**E**) were reactivated using NaBut. Expression of lytic transcript *RTA* was analyzed by qRT-PCR at 24 or 48 hrs post reactivation. See also figure S2C&D; Error bars (SD) are derived from triplicates. (**F**) Supernatant from cells in (E) was concentrated and 50 µl were used to quantify total KSHV genome copies (using LANA specific primers) by qPCR. (**G**) Serial dilutions of viral supernatant from (F) were used to infect 3×10^5^ WT HFF cells. Cells were trypsinized and passaged once to remove any uninternalized virus prior to harvesting total gDNA. qPCR was used to quantify average KSHV genome copies per HFF cell (LANA/AID gDNA). Plotted are triplicate averages fitted by linear regression. (**H**) shRNA was used to knock-down expression of UNG2 in empty vector control or AID-expressing BCBL-1 cells. 1 wk post selection cells were reactivated with NaBut for 24 and 48 hrs and analyzed for induction of *RTA* by qRT-PCR. (**I**) BCBL-1 cells were first stably transduced with vector overexpressing UNG2 or empty control, then with AID or empty vector control as in (A). At 6 wks post selection the four stable cell lines were reactivated with NaBut and assessed for infectivity same as in (G).

Deamination activities of host APOBEC proteins have previously been shown to directly inactivate retroviruses and Hepatitis B virus (HBV) as a form of innate antiviral defense [25]. To explore whether human AID activity can negatively impact KSHV fitness, we assessed viral latency and reactivation upon exposure to AID. To this end, we stably transduced BCBL-1 cells, a KSHV latently-infected cell line established from a human primary effusion lymphoma, with either AID or an empty vector control (Figure 3A). It should be noted that like most cell lines BCBL-1 cells do not express endogenous AID [44] and flow cytometric staining seen with vector control cells likely represents background intracellular staining. The two cell lines exhibited similar viability and proliferation rates (Figure S2A & B). To investigate a potential impact of AID on latency, we quantified levels of LANA, a latent transcript constitutively expressed in latently infected cells, in both cell lines as late as 12 weeks post selection. We observed similar levels of LANA transcript and protein (Figures 3B & C), which suggested that the latent virus is equivalently maintained in cells expressing AID and in cells expressing the empty vector.

Next, we treated the two stable cell lines with HDAC inhibitor sodium butyrate (NaBut) to stimulate lytic replication and compared induction of lytic transcripts. The master regulator of the latent-lytic switch, replication and transcription activator (RTA) is an immediate-early gene previously shown to be induced within hours of NaBut treatment [45]. Cells recently transduced with AID showed a modest impairment in reactivation as indicated by a decreased induction of RTA transcript relative to vector control at 48 hrs post-stimulation (Figure 3D). Interestingly, longer AID expression (up to 10 weeks after selection) led to a much more severe reactivation defect (Figure 3E). This defect was evident as early as 24 hrs post-stimulation. Assessment of RTA expression at four weeks post AID transduction revealed an intermediate phenotype (Figure S2C). Comparison of untreated to NaBut-treated samples further demonstrated that AID-expressing cells are able to induce lytic transcripts, but to a lesser extent relative to control (Fig. S2C). A similar expression pattern was observed when additional lytic transcripts were analyzed (Figure S2D). To ensure that the observed defect was not due to a random drift during an extended time in culture, we generated additional independent transductants and verified that they too exhibit a reproducible AID-dependent reactivation defect over time in culture (Figure S2E).

To explore whether the inhibition of lytic gene expression culminates in reduced viral titers, we quantified viral output in the supernatant of reactivated AID and control cells. Consistent with reduced *RTA* expression shown in figure 2E, AID-expressing BCBL-1 cells exhibited a roughly 3-fold reduction in the total virus output in the supernatant as measured by qPCR for LANA gDNA (Figure 3F). We then examined the ability of virus-containing supernatant to infect KSHV-negative fibroblasts, HFF cells. Interestingly, infection with equivalent volumes of supernatant from control and AID-expressing BCBL-1 cells uncovered a much greater disparity between the infectivity of the two viruses. Specifically, supernatant from AID-expressing cells yielded nearly 3 logs fewer intracellular KSHV genome copies per HFF cell (Figure 3G). Likewise, immunofluorescence assay revealed that while the supernatant from the control cell line resulted in robust infection of HFF cells as measured by LANA staining, supernatant from AID expressing cells produced dramatically fewer characteristic LANA nuclear speckles and also exhibited an overall reduction in LANA-positive cells indicative of a considerably reduced infectious viral titer (Figure S2F). Together these results reveal that AID-expressing cells not only secrete less total virus upon reactivation, but that this virus is substantially less infectious.

We next, examined whether AID's abundance would inversely correlate with KSHV infectivity. To this end, we stably transduced BCBL-1 cells with either a negative control shRNA or shRNA specifically designed to target AID, followed by a transduction with AID or empty vector control. We assayed the four resulting cell lines for their relative AID expression by flow cytometry and verified the efficacy of the knock-down with the anti-AID shRNA (Figure S2G). After 4 weeks in selection, these cell lines were reactivated and their supernatants used to infect fresh HFF cells. As evident in figure S2H, AID-expressing cells again exhibited a reduction in infectivity. Interestingly, the cell line with shRNA-mediated reduction of AID expression displayed an intermediate phenotype: a roughly two-fold greater infectivity than in the negative control shRNA plus AID cell line, and two fold less infectivity as compared to the vector control cell lines. Together these data demonstrated that although latent viral episome maintenance is not impacted by AID expression, viral reactivation and infectivity are negatively affected by host AID. Moreover, the resulting defect is proportionate both to AID expression level and to the length of exposure.

### UNG2 is required in AID-expressing KSHV infected B cells to allow for robust reactivation

Our observation that defective reactivation of KSHV generally occurs upon prolonged exposure of the virus to AID suggests that in the short-term this enzyme either has a minimal impact on KSHV reactivation or that KSHV has evolved mechanisms to partially evade AID-induced damage. A previous report looking at host proteins interacting with KSHV LANA identified Uracil DNA Glycosylase 2 (UNG2) as one of the factors directly recruited to the viral genome [46]. UNG2 is responsible for removing uracil residues generated upon either misincorporation of dUMP during replication, or deamination of cytosines [47], [48]. Thus, we hypothesized that UNG2 activity may offset the effect of AID on viral fitness. To test this possibility, we assessed the expression levels of *LANA* and *RTA* to reflect KSHV latency and reactivation capacity, respectively, upon knockdown of UNG2 via a previously validated shRNA [46]. The level of *LANA* remained unchanged in AID-expressing cells relative to control cells upon UNG2 knock-down (data not shown). In contrast, *RTA* expression upon NaBut stimulation was dramatically inhibited in AID-expressing cells transduced with UNG2 shRNA but not in control cells (Figure 3H). This effect was measurable as early as one week post-transduction with AID and comparable to, or even greater than that observed after a ten-week exposure to AID alone.

We next assayed whether overexpression of UNG2 in the presence of AID can help protect the virus from developing a defect in reactivation. To this end, we stably transduced BCBL-1 cells with UNG2, followed again by transduction with AID or empty vector control. Overexpression of UNG2 did not alter reactivation of AID-expressing cells when assayed by qRT-PCR (data not shown). However, we did observe a small (roughly two-fold) but reproducible rescue in infectivity in AID plus UNG2 overexpressing cells relative to AID-expressing cells with WT levels of UNG2 (Figure 3I). Given the relatively minor rescue in infectivity upon UNG2 overexpression, these results strongly suggest that the endogenous levels of UNG2 in BCBL-1 cells are largely sufficient in offering short-term protection of viral fitness. Taken together, these results demonstrate that UNG2 expression is required in KSHV infected cells to counteract AID activities and to allow for efficient reactivation and infectious virus output.

### KSHV-encoded miRNAs target 3′UTR of host AID

Many herpesviruses encode multiple independent effectors that target a particular host antiviral pathway in order to achieve efficient immune evasion [49]. We thus hypothesized that, instead of relying solely on the host UNG2, KSHV may have evolved alternative strategies to counteract AID activity. In the absence of infection, AID expression, stability and activity are endogenously regulated via multiple mechanisms. In particular, several endogenous miRNAs, miR-155, miR-181b and miR-93 have recently been shown to regulate AID at the level of translation via interaction with its 3′UTR [50]–[53]. Interestingly, EBV, another member of the human γ-herpesvirus family, has been shown to upregulate miR-155 expression anywhere from twenty to several thousand-fold depending on the cell type [54]–[56]. However, we observed little to no upregulation of the three host miRNAs known to regulate AID in the day-3 KSHV infected tonsillar B cells (Figure S3). Thus, we instead turned our attention to the cluster of KSHV-encoded miRNAs as potential AID modulators; especially given that one of these, miR-K12-11, is a bona fide ortholog of hsa-miR-155 [57], [58].

**Figure 4:**
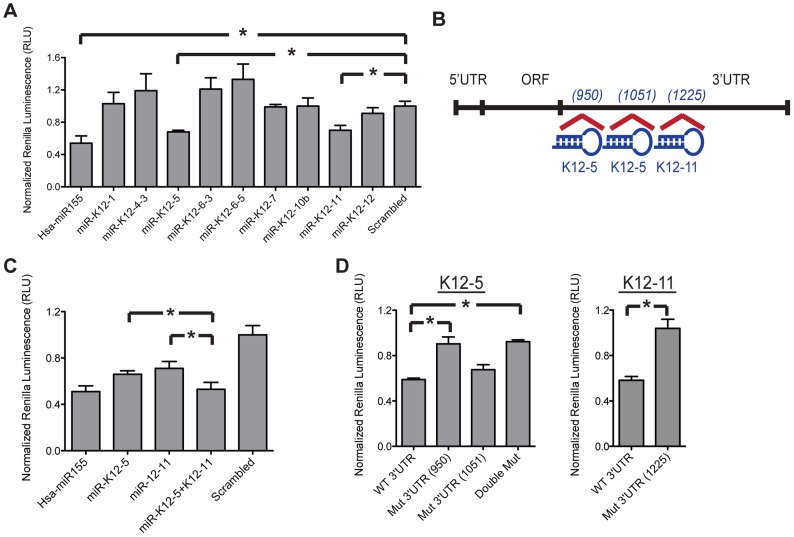
KSHV-encoded miRNAs directly target 3′UTR of AID. (**A**) HEK-293 cells were co-transfected with AID 3′UTR dual luciferase reporter and the indicated miRNA mimic or scrambled negative control miRNA. Renilla luciferase activity was normalized to firefly luciferase activity and then normalized to the average activity of the empty control reporter. Error bars (SD) are derived from triplicates. Scrambled control is set to 1, while miR-155 serves as a positive control. Shown is one representative experiment out of three performed. (**B**) A schematic representation of the full length *AID* mRNA and the predicted binding sites for miR-K12-5 and miR-K12-11; specific 3′UTR locations noted in prentices. (**C**) Relative AID 3′UTR luciferase reporter activity comparing transfection of a single miRNA, miR-K12-5 or miR-K12-11 versus both miRNAs, each at half of the original concentration. (**D**) Comparison of relative luciferase activity between WT and mutant 3′UTR AID reporters. Double mut combines mutations of mut 3′UTR (950) and (1051). Each bar represents fold change in reporter activity relative to scrambled control. Left panel represents co-transfection of reporter with miR-K12-5, right panel – with miR-K12-11. Statistically significant differences are indicated (*p<.01 by two-tailed, paired student t-test).

Bioinformatic analysis of seventeen mature KSHV miRNAs yielded nine candidates with partial sequence complementarity to the 3′UTR of AID mRNA. Table 1 ranks the nine candidates in order of their predicted strength of interaction, and describes the extent and type of nucleotide pairing between miRNA seed region and target 3′UTR according to Bartel [59]. To verify the validity of this prediction, we co-transfected miRNA mimics corresponding to the nine hits together with a dual reporter construct encoding a luciferase gene upstream of the *AID* 3′UTR and measured luciferase activity. We observed that similar to the positive control hsa-miR-155, expression of miR-K12-11 and miR-K12-5 mimics caused a significant decrease in luciferase signal, whereas none of the other miRNA mimics had a comparable effect (Figure 4A). This phenotype was specific to *AID* 3′UTR as the data were normalized to the luciferase signal in cells co-transfected with miRNAs and the control vector, which does not encode AID 3′UTR. Within the 3′UTR of *AID*, miR-K12-5 is predicted to bind at two adjacent positions that are located slightly upstream of the predicted miR-K12-11 binding site (Figure 4B). During viral latency, all KSHV-encoded miRNAs are generated from a single-stranded, primary, polycistronic transcript [60]. Consequently, we examined whether co-expression of both miR-K12-11 and miR-K12-5 results in an additive effect on AID reporter downregulation. Upon co-transfection of the two miRNA mimics, we observed an even greater reporter inhibition relative to what is achieved with either miRNA alone (Figure 4C). To verify specificity of the interaction between *AID* and miR-K12-5 or miR-K12-11, we mutated seven to eight residues within the 3′UTR of *AID* predicted to interact with the seed regions of miRNAs K12-5 and K12-11 (see table S1 for sequences of mutant constructs). As expected, these specific mutations rescued reporter expression further validating our bioinformatics analysis (Figure 4D). In the case of miR-K12-5, the first of the two predicted binding sites makes the greatest contribution to reporter inhibition, which correlates with the corresponding strength of interaction score in Table1.

**Table 1:** Bioinformatic Analysis of KSHV miRNAs binding to human AID.

miRNA	Hit-position	Site Type	Score
miR-K12-11	1225	m8	1.252807
miR-K12-5	950	m8	1.212495
	1051	m6	1.053520
miR-K12-6-5	717	m7_A1	1.117598
miR-K12-4-3	1107	m6	1.048571
	766	m6	1.038917
miR-K12-6-3	1211	m7_M8	1.029981
miR-K12-10b	1473	m6	1.019462
miR-K12-7	2231	m6	1.012112
miR-K12-1	1989	m7_M8	.974461
	1854	m7_M8	.966365
miR-K12-12	1919	m6	.968706
	1770	m6	.963473
	2085	m6	.960405

Columns identify miRNA name, position within AID mRNA where predicted binding of seed sequence occurs, type of target site and miRNA:gene interaction score according to Bartel D.P. (2009). miRNAs are ordered according the score, from strongest to weakest predicted interaction.

### miR-K12-5 and miR-K12-11 target full length AID for downregulation when expressed at physiological levels

**Figure 5:**
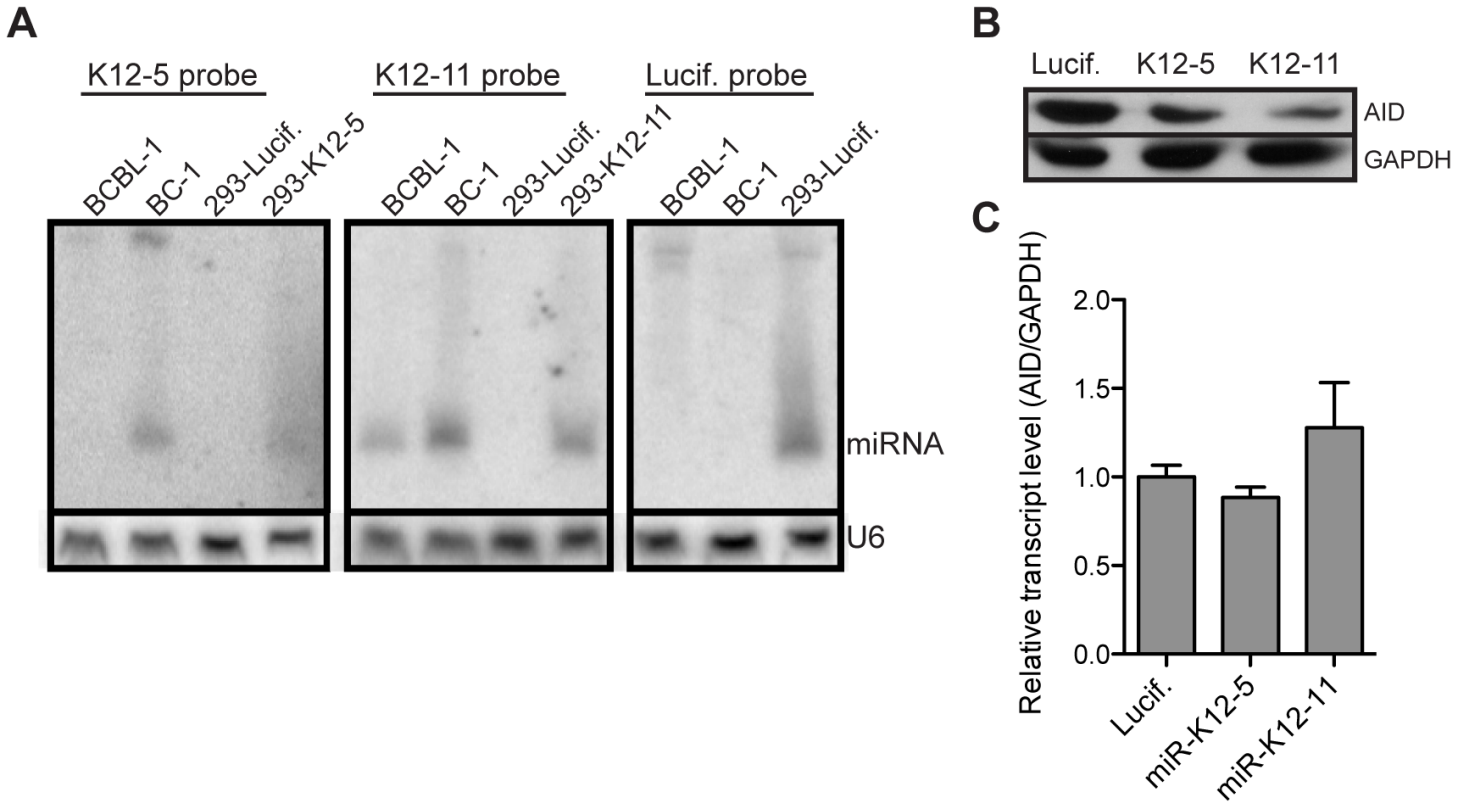
miR-K12-5 and miR-K12-11 target full length AID for downregulation when expressed at physiological levels. (**A**) HEK-293 cells were stably transduced with shRNA constructs expressing individual indicated KSHV miRNAs or negative control miRNA. Total RNA was harvested from the stable cell lines or BC-1 and BCBL-1 cell lines latently infected with KSHV for Northern blot analysis of miRNA expression. Probe binding to miR-K12-5, miR-K12-11 or siRNA against luciferase gene is indicated above each panel. U6 expression serves as a loading control. (**B**) HEK-293 cells were stably transduced with full length *AID* mRNA along with the indicated shRNA construct. Total protein was harvested and AID expression detected by Western blot. GAPDH serves as a loading control. (**C**) Total RNA was isolated from the same cells as described in (B). Relative *AID* expression was quantified by qRT-PCR analysis. Error bars (SD) are derived from triplicates.

To confirm our luciferase reporter results in a more physiologically relevant system, we cloned miR-K12-11 and miR-K12-5 into a short hairpin retroviral construct that would require processing by the endogenous machinery to yield functional mature miRNAs. As a negative control we generated a construct encoding an shRNA designed to target the luciferase gene. Because most cell lines do not express endogenous AID, we generated HEK-293 cells that stably express full-length *AID* mRNA containing the natural 5′ and 3′ UTRs. Subsequently, we transduced these AID-expressing HEK-293 cells with our miRNA constructs. A northern blot was performed on transduced cells to verify that miR-K12-5 and miR-K12-11 were expressed at physiological levels. BCBL-1 and BC-1, two cell lines derived from KSHV infected patients, served as controls. While expression levels of both miRNAs varied somewhat between the two control cell lines, which is consistent with a previous report [60], expression levels of these miRNAs in HEK-293 cells were comparable with at least one of the two control cell lines (Figure 5A). Next, we assessed the level of AID protein in the presence of either miR-K12-5 or miR-K12-11. Consistent with our reporter assay, expression of both viral miRNAs resulted in diminished levels of AID protein (Figure 5B). Quantitative RT-PCR analysis revealed that neither of the miRNAs destabilized the *AID* transcript (Figure 5C), suggesting that inhibition is occurring at the level of translation, as is the case with endogenous miRNAs.

### KSHV miRNAs mediate downregulation of AID in the context of the viral genome

**Figure 6:**
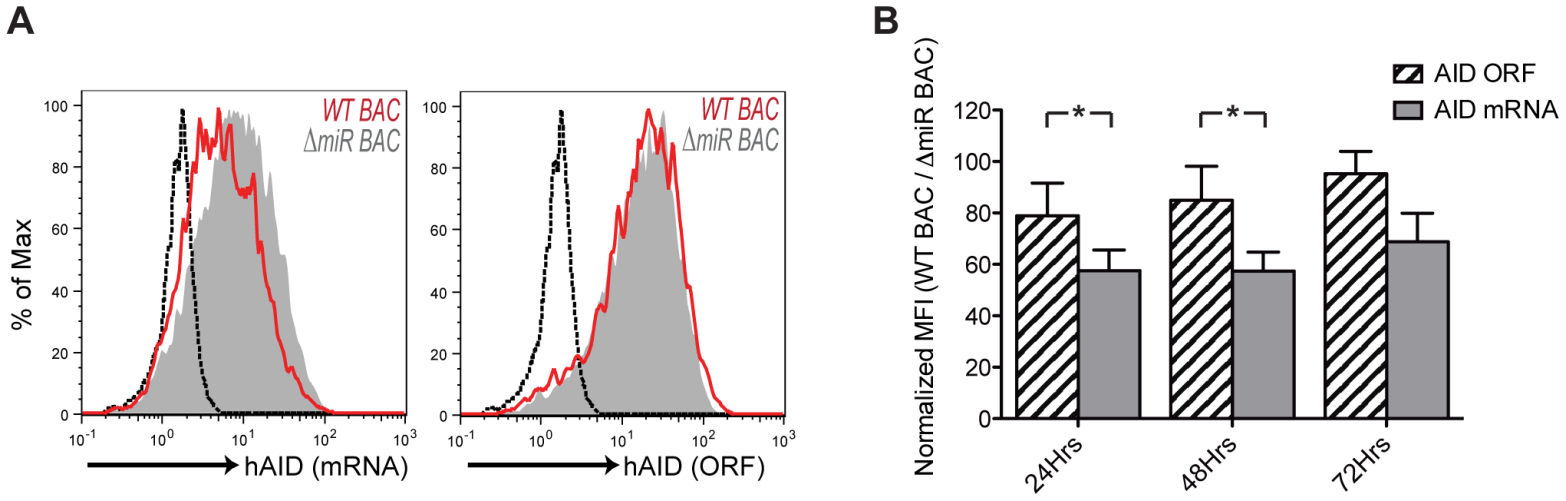
KSHV miRNAs downregulate AID in the context of the entire viral genome. (**A**) HEK-293 cells were stably transduced with constructs encoding full length *AID* mRNA (hAID mRNA) or open reading frame of *AID* (hAID ORF) and transiently transfected with either WT KSHV BAC (red) or BAC lacking miRNA cluster, ΔmiR BAC(filled gray). Intracellular AID expression was analyzed by flow cytometry at 48 hrs post transfection. Dashed black histogram reflects background staining of AID-negative WT HEK-293 cells. (**B**) Quantification of AID downregulation upon WT KSHV BAC transfection relative to ΔmiR BAC at 24, 48 or 72 hrs post transfection. Data represent at least three independent experiments for each time point. Dashed bars represent cells stably expressing hAID ORF, solid gray bars represent cells stably expressing hAID mRNA. The percentage of AID downregulation (% MFI), in cells transfected with WT KSHV BAC was calculated relative to cells transfected with the ΔmiR BAC after subtracting background MFI. Shown are mean values ± SD (*p<.05 by two-tailed, paired student t-test).

To investigate the contribution of KSHV miRNAs to the regulation of AID expression in the context of the entire viral genome, we utilized a recombinant GFP-tagged bacteria artificial chromosome (BAC) encoding the entire KSHV genome (WT BAC) or one lacking the miRNA cluster from its latency region (ΔmiR BAC) [61], [62]. While expression of these BAC's in mammalian cells does not yield high viral titers, transfection of these constructs into cells recapitulates gene expression seen upon infection with KSHV viral particles. We transiently transfected HEK-293 cells stably expressing AID with either WT or ΔmiR BAC and compared AID levels between the resulting GFP-positive cell fractions. AID protein expression was significantly lower in cells expressing full length *AID* mRNA that were transfected with WT BAC compared to cells transfected with ΔmiR BAC (Figure 6A). However, this difference was largely mitigated in cells expressing only the *AID* open reading frame (AID ORF), confirming that the KSHV miRNAs predominantly target the *AID* mRNA via its UTR. Given that the KSHV miRNA cluster may affect global host gene expression, we observed a minor miRNA cluster-dependent decrease in AID ORF expression at early time points following transfection (24 and 48 hrs) in a number of independent experiments (Figure 6B). Nonetheless, AID produced by the full-length transcript was significantly more downregulated compared to *AID* ORF. (Figure 6B). Together these results support the role of KSHV-encoded miRNAs in the regulation of AID protein levels via the 3′ UTR of *AID*.

## Discussion

Traditionally, AID is defined as a factor required for a robust humoral immune response given its role in SHM and CSR. Our study reveals an additional role for AID in innate immune defense against a human γ-herpesvirus, KSHV. Our data demonstrate that KSHV infection of naïve primary B cells leads to a rapid upregulation of AID in all cells harboring the virus, and that this elevated level of expression is sustained for the duration of primary cell culture. Although it is known that AID can be induced in a variety of tissues infected with viral and bacterial pathogens [27]–[30], the biological significance of this expression in relationship to the pathogen remains largely uncharacterized. We provide the first evidence that AID expression not only contributes to alerting the innate immune system to the pathogen through the induction of ligands for the activating NK cell receptors, but also directly regulates viral fitness by inhibiting its ability to undergo lytic reactivation and limiting the spread of infection. Ectopic AID induction has puzzled the field for some time given its ability to increase the risk of oncogenic transformation. Our data demonstrate that AID function imparts a significant advantage in immune defense against a viral pathogen and thus, shed light on the value of AID expression outside of GC B cells.

Pathogens previously reported to upregulate AID accomplish this through a variety of stimuli, which ultimately converge to activate NF-κB. HIV, for example, ligates CD40 receptors via virion-associated CD40L [27], while HTLV-1 and HCV do so via intracellular viral protein Tax and core proteins, respectively [28], [63]. In our case, vFLIP, latent viral gene with a known role in NF-κB activation is sufficient to induce AID expression in primary human B cells. We are currently working to generate a recombinant KSHV lacking vFLIP expression to investigate whether it is completely or partially defective in AID induction.

There is growing evidence for the importance of NK cells in controlling KSHV infection. KSHV positive PEL cell lines are preferentially lysed by NK cells from healthy blood donors when compared with KSHV negative lymphoma cell lines [64]. Similarly, NK cells specifically eliminate KSHV infected fibroblasts [65]. *In vivo* resolution of Kaposi's sarcoma directly correlates with restoration of NK cell activity [64]. We also observed an upregulation of stimulatory NK cell ligands in our primary human B cells upon KSHV infection. To counteract NK cell recognition, KSHV employs a variety of strategies to downregulate activating ligands during its latent and lytic replication programs. Viral ubiquitin ligase MIR2 and ORF54 can interfere with NK cell recognition by increasing turnover of MICA and MICB, and AICL, respectively [66], [67]. Additionally, miR-K12-7 has been shown to decrease levels of *MICB* mRNA [68]. This multi-faceted effort to escape recognition by NK cells highlights the importance of this cell type in controlling KSHV pathogenesis.

DNA double strand breaks that result from AID activity are known to trigger DNA damage stress response [69]. One of the downstream consequences of such responses is the upregulation of stimulatory NK cell ligands [38]. One study has linked genotoxic stress associated with AID function to the induction of the activating NK ligands in cells infected with a murine retrovirus, Ab-MLV [26]. *In vivo*, this response resulted in protection against Ab-MLV and improved animal survival. An analogous observation was made for APOBEC3G, where cytidine deamination enhanced NK cell recognition of HIV-infected cells via the DNA damage response pathway [39]. Current limitations in efficient knock-down of gene expression in primary cells have prevented us from establishing a direct link between KSHV-induced AID expression and NK ligand upregulation in our system. However, consistent with the previous reports, we have shown that KSHV infected cells upregulate NKG2D ligands in a DNA damage dependent manner, suggesting a possible role for AID in potentiating NK cell recognition of KSHV infected cells. The incomplete inhibition of ligand induction we observe with DDR inhibitors could be a result of partial rather than complete inhibition of the pathway. Furthermore, additional pathways, such as innate-immune sensor activation or virally induced hyper-proliferation of infected cells may contribute to ligand induction. Nonetheless, sustained AID expression during KSHV infection is likely to augment the overall surface expression of NK ligands given its ability to cause DNA damage.

Beyond this, we observe that expression of AID has a direct adverse impact on KSHV. Our data reveal that while latency is unaffected by AID expression within the time frame examined, lytic reactivation and infectivity are significantly suppressed. The fact that UNG2 knockdown in conjunction with AID expression enhances this phenotype supports the notion that the observed defect is due to deamination of DNA by AID. One possible explanation for the decrease in reactivation and an even more drastic drop in infectious virion production is accumulation of detrimental mutations within the virus. To address this, we sub-cloned and sequenced several one kilobase-long viral genomic regions isolated from virions secreted by reactivated cells expressing AID or vector control. We observed very few mutations and their frequency did not correlate with AID expression (data not shown). The result could be either a consequence of insufficient sequence coverage or isolation of KSHV virions, which may have biased our viral genome sampling toward the less mutated productive viral particles. Based on a recent study demonstrating the ability of AID to directly deaminate viral RNA in an *in vitro* model of HBV [70], it is also plausible that AID may edit KSHV RNA transcripts, thereby contributing to a defect in infectivity. Additionally, because KSHV relies on numerous host components, such as transcription factors and protein synthesis machinery, to initiate and drive lytic reactivation [71], [72], mutations within the host genome could have negatively impacted viral reactivation. Similar to viral genes, we did not uncover specific AID-dependent mutations within the host genes, although our sequence coverage may again have been insufficient to pinpoint such regions. Finally, AID expression may generate a cellular environment that is deleterious to KSHV reactivation. For instance, cellular pathways activated by AID (e.g. DDR) may affect the activity of host proteins known to contribute to RTA promoter activation and cooperation with RTA to initiate transcription of downstream viral lytic genes [72]. While latency is generally accepted as the default program of the virus, reactivation is thought to be important in reseeding and long-term persistence of the latent viral pool. Thus, protection against the detrimental effects of AID on lytic reactivation and infectious virion production is of vital importance to a persistent virus like KSHV.

The substrate of AID is ssDNA, which explains its preference for actively transcribed genes [73]. Silencing the vast majority of its genome during latency may thus be a deliberate evolutionary strategy of KSHV aimed at avoiding AID mutagenesis among other advantages. In fact, KSHV, as well as its murine homolog, MHV68, maintain latency until terminal differentiation of germinal center B cells into plasma cells [74], [75]. As part of this transition, B cells downregulate *AID* among other genes, and hence relieve the virus from potential mutational insult.

Previously characterized recruitment of UNG2 by LANA may serve as one mode of preservation of KSHV genome integrity in the presence of AID. In fact, KSHV is not the only virus employing such a strategy. HIV Vpr has been shown to counteract APOBEC3G by diminishing the incorporation of uridine through its interaction with UNG2 [39]. However, as evident from our reactivation data, even in the presence of wild type levels of UNG2, AID activity can be detrimental to KSHV in the long run. Our primary B cell infections revealed that AID expression is not transient, but sustained and increased in the course of an infection. Moreover, it is conceivable that upon exposure to pro-inflammatory cytokines and interaction with other activated immune cells during an *in vivo* infection, AID expression may be augmented even further. Hence, the modulation of AID levels or activity is of vital importance to the long-term success of KSHV.

The KSHV close relative, EBV, is known both to upregulate miR-155 as well as specifically inhibit AID via its latency protein EBNA2 [76]. While we have not observed analogous activity from KSHV, we uncovered two distinct viral miRNAs, miR-K12-5 and miR-K12-11, capable of decreasing AID protein expression. Such an approach can be particularly advantageous given that miRNAs can modulate expression of AID and numerous other targets without generating antigenic peptides, which may put the virus at risk for immune detection during latency. A partial decrease in AID protein level, achieved through expression of miR-K12-5 and miR-K12-11 is very consistent with the role of miRNAs in fine-tuning gene expression. Importantly, AID protein function is highly sensitive to its abundance given that AID exhibits haploinsufficiency for antibody diversification and chromosome translocations [77]. Additionally, AID expression levels correlate both with mutation rates in cell lines [78] and production of infectious KSHV titers in our study. In light of these findings, even a partial decrease in AID expression achieved by miR-K12-11 and miR-K12-5 may offer significant contribution to viral defense against AID.

The current body of literature largely supports the idea that latently expressed viral miRNAs actively promote latency [61], [79], [80], in part, through direct or indirect regulation of immediate early genes like RTA [81], [82]. At least one study, however, reported the opposite by demonstrating that inhibition of certain KSHV miRNAs reduced the percentage of cells undergoing spontaneous reactivation by about 40% [83]. Our data highlight another level of regulation by virally encoded miRNAs. Rather than preventing or promoting spontaneous reactivation, miRNA-dependent regulation of AID ensures that reactivation and propagation of KSHV are as efficient as possible upon receipt of appropriate reactivation stimuli.

Since the discovery of its catalytic function AID has been directly implicated in mutations and chromosomal translocations responsible for tumorigenesis [84]. In line with that knowledge, researchers who previously observed AID expression in non-GC B cells (e.g. hepatocytes, gastric epithelial cells) often labeled it as inappropriate. Our study supports the notion that AID can serve a protective role during viral pathogenesis by marking infected cells for elimination and inhibiting viral fitness. Therefore, AID induction in the context of oncogenic viruses such as KSHV may actually limit transformation rather than serve as the culprit.

## Materials and Methods

### Ethics statement

Fresh tonsillar tissues from routine tonsillectomies were obtained from the Cooperative Human Tissue Network. All tissues were anonymous pathological samples obtained according to a UC Berkeley Institutional Review Board-approved protocol.

### Tissue culture and B cell enrichment

Tonsillar tissues were minced and passed through a 40-µm cell strainer. Mononuclear cells were isolated by centrifugation over a Histopaque (Sigma) cushion at 1400× *g* for 15 min and cultured in medium consisting of RPMI 1640 supplemented with 15% FBS, 1% pen-strep, 1 mM sodium pyruvate, 1% nonessential amino acids (Mediatech), 2 mM L-glutamine, and 1% fungizone (Invitrogen). Prior to sorting of infected cells, B cells were enriched using EasySep Human CD19 Positive Selection Kit (Stem Cell Technologies) following manufacturer's instructions. HEK-293, HFF and iSLK.219 cells were cultured in DMEM, 10% FBS and 1% pen-strep. BCBL-1 cells were cultured in RPMI 1640, 10% FBS and 1% pen-strep. NK-92 cells were cultured in alpha-MEM, 10% FBS, 10% Horse serum, 0.1 mM 2-mercaptoethanol, 0.02 mM folic acid, 200 U/ml recombinant IL-2 and 1% pen-strep.

### KSHV reactivation and infections

For primary tonsillar cell co-culture infections, iSLK.219 cells grown to 70% confluency were reactivated with 0.2 µg/ml doxycycline and 1 mM sodium butyrate (NaBut) for 24–48 hrs, tonsillar cells were added into the same flask and infection was allowed to proceed for indicated amount of time. Virus production from BCBL-1 cells was performed by reactivating cells at 5×10^5^ cells/ml with 0.3 mM NaBut for 5 days. Virus-containing supernatant was filtered through .45 um filter, concentrated 50× by centrifugation at 24,000× *g* for 2 hr. Pelleted virus was resuspended in PBS and used for viral gDNA isolation or infection. WT HFF cells were infected via 2 hr spinfection at 1,000× *g* with addition of polybrene (4 µg/ml).

### Constructs and reagents

vFLIP was cloned into pMax vector (Lonza) and co-transfected with pMax-GFP at a 4∶1 ratio for gating on positively transfected primary cells. AID was cloned into pQC-X-IN vector with an N-terminal 3×Flag-tag for expression in HEK-293 cells, or without a tag into pCru-X-IP retroviral vector for expression in BCBL-1 cells. UNG2 was cloned into pQC-X-IN vector for overexpression in BCBL-1 cells. Hsa-miR-155, KSHV miRNAs, and anti-luciferase shRNA were cloned into MSCV-miR-30 based expression system as previously described [57], [85]. shRNA's were cloned into pLKO.1-hygro vectors for stable knock down of target gene. The 3′ UTR of AID was cloned into dual luciferase reporter vector psiCHECK-2 (Promega). Mutant 3′UTR reporters were generated by introducing 7–8 nucleotide substitutions within the regions predicted to bind seed sequence of the corresponding miRNA (see Table 1S for mutant sequences). The anti-UNG2 shRNA sequence was adopted from a previously published study [46]. Retroviral transductions were performed by transfecting vector of interest into the Phoenix retroviral packaging cell line along with a plasmid encoding vsv-g, 48 hrs later filtering the resulting supernatant and spinfecting the target cells with the supernatant for 2 hrs at 1,000× *g* with addition of polybrene (4 µg/ml). 24 hr post transduction cells were selected with either 600 µg/ml G418 or 1 µg/ml puromycin. Selection was complete within 5–7 days post-transduction, gene or protein expression verified and cells maintained in the presence of drugs for the duration of culture. KSHV BAC constructs were a kind gift from the Gao group [62]. All transfections of cell lines were performed using Lipofectamine2000 (Invitrogen). Primary cells were transfected using Amaxa nucleofection protocol U-015 (Lonza) using DNA purified with EndoFree Plasmid Maxi Kit (Qiagen). Each gene-expressing vector was mixed and co-transfected with pMax-GFP transfection control at 4∶1 ratio. Transfection efficiency varied between 2–10% as measured by percent GFP-expressing cells.

### Flow cytometry, cell sorting and cytotoxicity assay

Primary cells were pre-blocked with Anti-Human CD32 Blocker (Stem Cell Technologies). For cell surface marker staining, cells were washed and stained in 1% FBS in PBS with anti-hULBP-2/5/6 and anti-MICB (R&D Systems), anti-Human CD19 PerCP-Cy5.5 (ebiosciences), anti-Flag M2 (Sigma-Aldrich) antibodies. For intracellular AID staining, cells were fixed, permeabilized and stained with anti-AID antibody (Millipore) using BD Cytofix/Cytoperm Fixation/Permeabilization Solution Kit (Thermo Fisher) following the manufacturer's instructions. Cells were analyzed using the LSR Fortessa cell analyzer (BD Biosciences) or sorted on MoFlo high-speed sorter (DakoCytomation).

Susceptibility of infected cells to NK-mediated lysis was evaluated via a flow-cytometry based cytotoxicity assay. Briefly, KSHV infected or uninfected cells were sorted according to GFP expression. Uninfected cells were stained with CFSE to distinguish them from the effector cells, while virally infected were distinguished based on GFP expression. Targets were co-cultured with different ratios of NK92 effector cells for 5 hours at 37°C. Cells were then washed and stained with the LIVE/DEAD® Fixable Violet Dead Cell Stain kit (Invitrogen) for 30 min on ice. Cells were washed twice and analyzed on a LSR Fortessa.

### Immunofluorescence staining

Cells were washed and allowed to adhere onto polylysine treated glass slides. Samples were fixed/permeabilized in 50% acetone/50% methanol at −20 C. Cells were rehydrated in 3% BSA, 1% glycine in PBS, and stained with anti-LANA antibody (Advanced Biotechnologies) followed by anti-rat FITC in 3% BSA in PBS buffer in a humidified chamber. Washes were done with 4% Tween-PBS. Slides were imaged with 40× lens using a fluorescent deconvolution microscope.

### Quantitative real-time PCR

RNA was extracted in Trizol (Invitrogen), treated with RQ1 DNase (Promega), and total RNA was reverse transcribed using oligo(dT)_15_ primer (Integrated DNA Technologies) and SuperScriptII (Invitrogen) at 42°C for 50 minutes. gDNA for genome copy quantification was isolated using DNeasy blood and tissue kit (Qiagen). cDNA and gDNA templates were analyzed using iTaq SYBR Green Supermix With ROX (BioRad) on an ABI7300 Real Time PCR System. GAPDH was used to normalize mRNA expression, amplification of AID genomic DNA was used to calculate viral genome copies per host cell. Standard curve for absolute KSHV genome copy calculations was set up with serial dilutions of LANA expressing plasmid. For miRNA expression analysis total RNA was reverse-transcribed using cDNA Synthesis kit for miRNA (OriGene). miRNA expression was assessed using miRNA-specific forward and universal reverse primers.

### Luciferase assay

HEK-293 cells were co-transfected with 2.5 pmol miRNA mimics (Qiagen) and 50 ng of reporter DNA construct per well of a 96-well plate in triplicates. 24 hrs later cells were lysed in Passive Lysis Buffer (Promega) and luciferase activity measured using an LMAXII^384^ luminometer (Molecular Devices). Renilla luciferase signal was used as the primary reporter gene, while Firefly luciferase was used to control for well-to-well transfection variation.

### Northern blot

Thirty micrograms of total RNA was loaded onto 15% 8 M urea polyacrylamide gel and transferred onto Hybond-N+ Nucleic acid transfer membrane (Amersham) following electrophoresis. Probe labeling was performed using T4 polynucleotide kinase (New England Biolabs) in the presence of [γ^32^-P]ATP.

### Western blot

Cells were lysed in RIPA buffer (50 mM Tris [pH 8], 150 mM NaCl, 1% Nonidet P-40, 0.5% sodium deoxycholate, 0.1% SDS, and a protease inhibitor cocktail (Roche)). The lysates were cleared of debris by centrifugation in a microcentrifuge at 4°C and protein concentration quantified by BCA assay (Pierce). Samples were run on a 12% polyacrylamide gel and transferred to a polyvinylidene difluoride (PVDF) membrane. Blots were probed with anti-FLAG M2 (Sigma-Aldrich) and anti-GAPDH (Abcam) antibodies.

### Cell proliferation assays

BCBL-1 stable transductants were counted and plated at 4×10^5^ cells/ml in individual wells of a 12-well plate in replicates. 24 hrs later cells in each well were re-counted and doubling time determined. Additionally, BCBL-1 stable transductants were lab**e**led with Cell Proliferation Dye eFluor 670 (eBioscience) per manufacturer's instruction. At indicated time points, dilution of the dye, and hence, proliferation was assessed by flow cytometry.

### Statistical analysis

A two-tailed, paired student t-test was performed on all samples where statistical significance is indicated.

## Supporting Information

Figure S1 **Enrichment of CD19+ tonsillar cells.** KSHV infected primary tonsillar cells were enriched for B cells using CD19 positive selection kit. Total tonsillar cells were analyzed for CD19 expression by flow cytometry pre-enrichment (filled gray) and post-enrichment (red). Black dashed histogram represents isotype control staining.
(TIF)

Figure S2 **Prolonged exposure of KSHV to AID results in lytic reactivation and infectivity defect in BCBL-1 cells.** (**A**) BCBL-1 cells stably expressing AID or empty vector control at 10 wks post selection were plated in individual wells at 4×10^5^ cells/ml. 24 hrs later cells were re-counted and doubling time calculated. Error bars (SD) are derived from n = 6. (**B**) BCBL-1 cells stably expressing AID (blue) or empty vector control (red) were labeled with Proliferation Dye eFluor 670. At 1, 2 or 4 days post labeling cells were analyzed by flow cytometry for dilution of the dye/proliferation. Filled gray histogram represents unlabeled cells. (**C**) BCBL-1 cells stably transduced with empty vector control or AID for 4 wks were left untreated or reactivated using NaBut. Expression of lytic transcript *RTA* was analyzed by qRT-PCR either without treatment or at 24 and 48 hrs post reactivation. (**D**) BCBL-1 cells stably expressing AID or empty vector control at 10 wks post selection were reactivated using NaBut. Expression of lytic transcripts K1 and K8.1 was analyzed by qRT-PCR at 24 or 48 hrs post reactivation. Error bars (SD) are derived from triplicates. (**E**) BCBL-1 cells transduced independently from cell lines presented in figure 2 were analyzed by qRT-PCR for the expression of *RTA* after 48 hr NaBut treatment. Shown is time course analysis from 1 wk to 10 wks post transduction. Error bars (SD) are derived from triplicates. (**F**) Equal numbers of BCBL-1 cells stably expressing AID or empty vector control (same as presented in fig. 3E–G) were reactivated for 5 days and equal volumes of supernatant used to infect WT HFF cells. Staining of HFF cells for KSHV protein LANA (green) and DAPI (blue) reflects relative infectious particles in each supernatant. (**G**) BCBL-1 cells were first transduced with either negative control shRNA or anti-AID shRNA, then each was also transduced with AID or empty vector control. The four resulting cell lines were analyzed for intracellular AID expression by flow cytometry upon completion of selection. Dashed black histogram represents unstained control. (**H**) At 4 wks post selection cells described in (G) were reactivated with NaBut for 4 days, and resulting supernatants were assessed for infectivity same as in Figure 2G.
(TIF)

Figure S3 **KSHV infection does not dramatically upregulate expression of endogenous miRNA regulating AID.** Primary tonsillar cells were infected with KSHV by co-culture with reactivated iSLK.219 cells. After day 3 of co-culture infected, GFP+ and uninfected, GFP− B cells were sorted and total RNA harvested. Relative expression of *miR-93*, *miR-155* and *miR-181b* was assessed via qRT-PCR analysis. Presented is fold induction of miRNA in infected relative to uninfected cells. Data are normalized to the expression of miR-191. Error bars (SD) are derived from triplicates. Shown is one representative experiment out of three performed.
(TIF)

Table S1 **Sequences of DNA oligos used in experimental procedures.** The table contains DNA sequences for primers and probes used for each indicated gene. The application is specified in column two. When applicable Fwd refers to the forward primer, Rev refers to the reverse primer.
(DOCX)

Text S1 **Supporting materials and methods.**
(DOCX)
