# Supplementary figures and images for: A Role for Host Activation-Induced Cytidine Deaminase in Innate Immune Defense against KSHV
Source: PLoS Pathog. 2013 Nov 7;9(11):e1003748. doi: 10.1371/journal.ppat.1003748 (PMC3820765; doi:10.1371/journal.ppat.1003748)

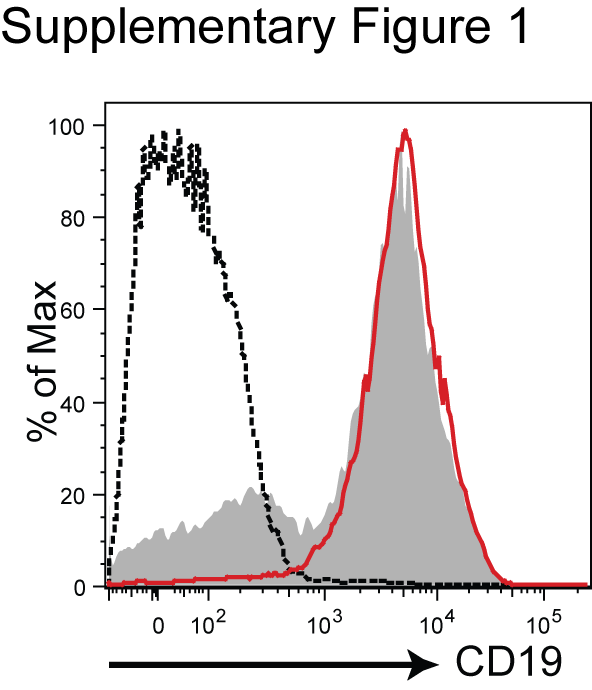

Supplement: Figure S1 — Enrichment of CD19+ tonsillar cells. KSHV infected primary tonsillar cells were enriched for B cells using CD19 positive selection kit. Total tonsillar cells were analyzed for CD19 expression by flow cytometry pre-enrichment (filled gray) and post-enrichment (red). Black dashed histogram represents isotype control staining. (TIF) [file ppat.1003748.s001.tif]

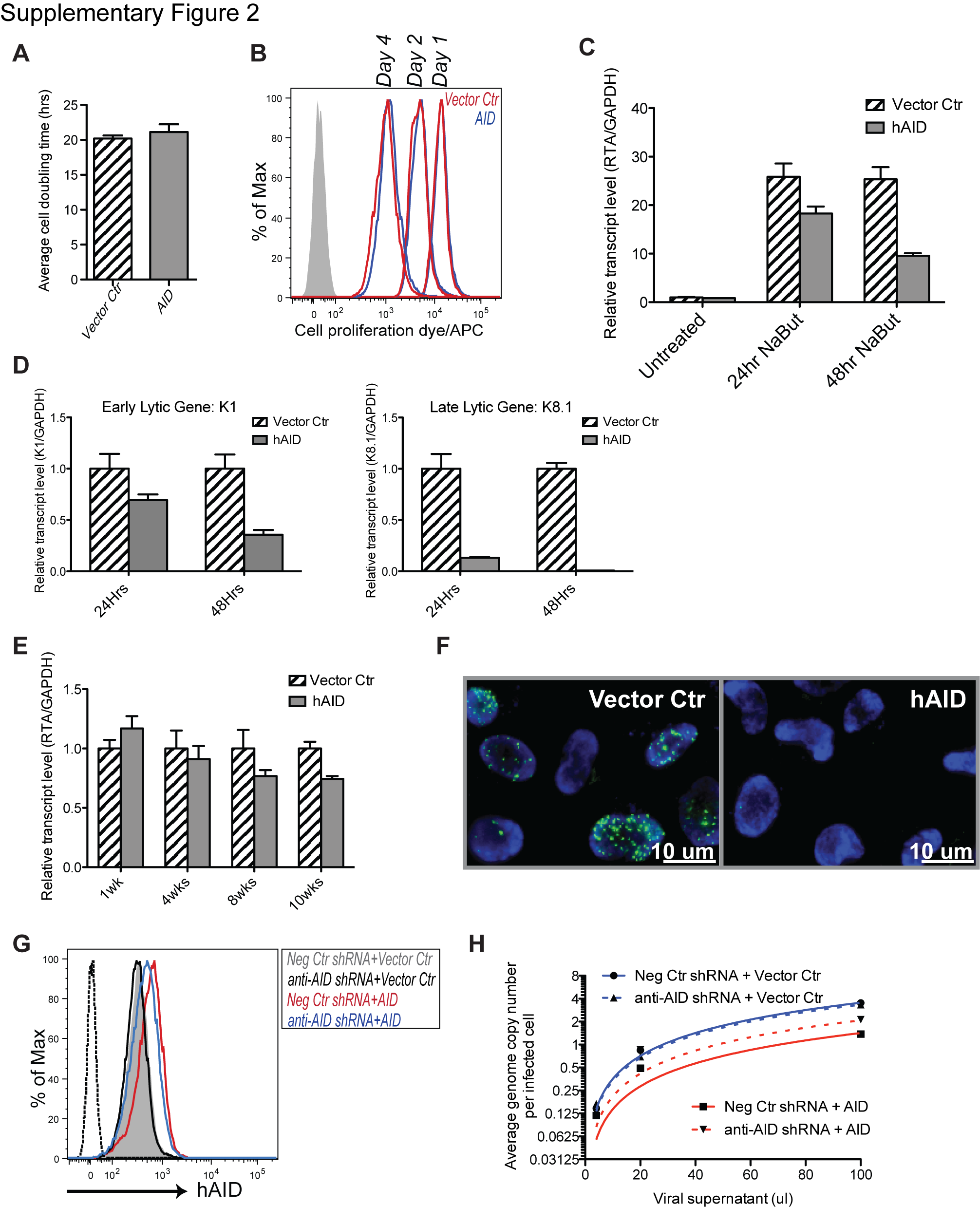

Supplement: Figure S2 — Prolonged exposure of KSHV to AID results in lytic reactivation and infectivity defect in BCBL-1 cells. (A) BCBL-1 cells stably expressing AID or empty vector control at 10 wks post selection were plated in individual wells at 4×10^5^ cells/ml. 24 hrs later cells were re-counted and doubling time calculated. Error bars (SD) are derived from n = 6. (B) BCBL-1 cells stably expressing AID (blue) or empty vector control (red) were labeled with Proliferation Dye eFluor 670. At 1, 2 or 4 days post labeling cells were analyzed by flow cytometry for dilution of the dye/proliferation. Filled gray histogram represents unlabeled cells. (C) BCBL-1 cells stably transduced with empty vector control or AID for 4 wks were left untreated or reactivated using NaBut. Expression of lytic transcript RTA was analyzed by qRT-PCR either without treatment or at 24 and 48 hrs post reactivation. (D) BCBL-1 cells stably expressing AID or empty vector control at 10 wks post selection were reactivated using NaBut. Expression of lytic transcripts K1 and K8.1 was analyzed by qRT-PCR at 24 or 48 hrs post reactivation. Error bars (SD) are derived from triplicates. (E) BCBL-1 cells transduced independently from cell lines presented in figure 2 were analyzed by qRT-PCR for the expression of RTA after 48 hr NaBut treatment. Shown is time course analysis from 1 wk to 10 wks post transduction. Error bars (SD) are derived from triplicates. (F) Equal numbers of BCBL-1 cells stably expressing AID or empty vector control (same as presented in fig. 3E–G) were reactivated for 5 days and equal volumes of supernatant used to infect WT HFF cells. Staining of HFF cells for KSHV protein LANA (green) and DAPI (blue) reflects relative infectious particles in each supernatant. (G) BCBL-1 cells were first transduced with either negative control shRNA or anti-AID shRNA, then each was also transduced with AID or empty vector control. The four resulting cell lines were analyzed for intracellular AID expression by flow cytom [file ppat.1003748.s002.tif]

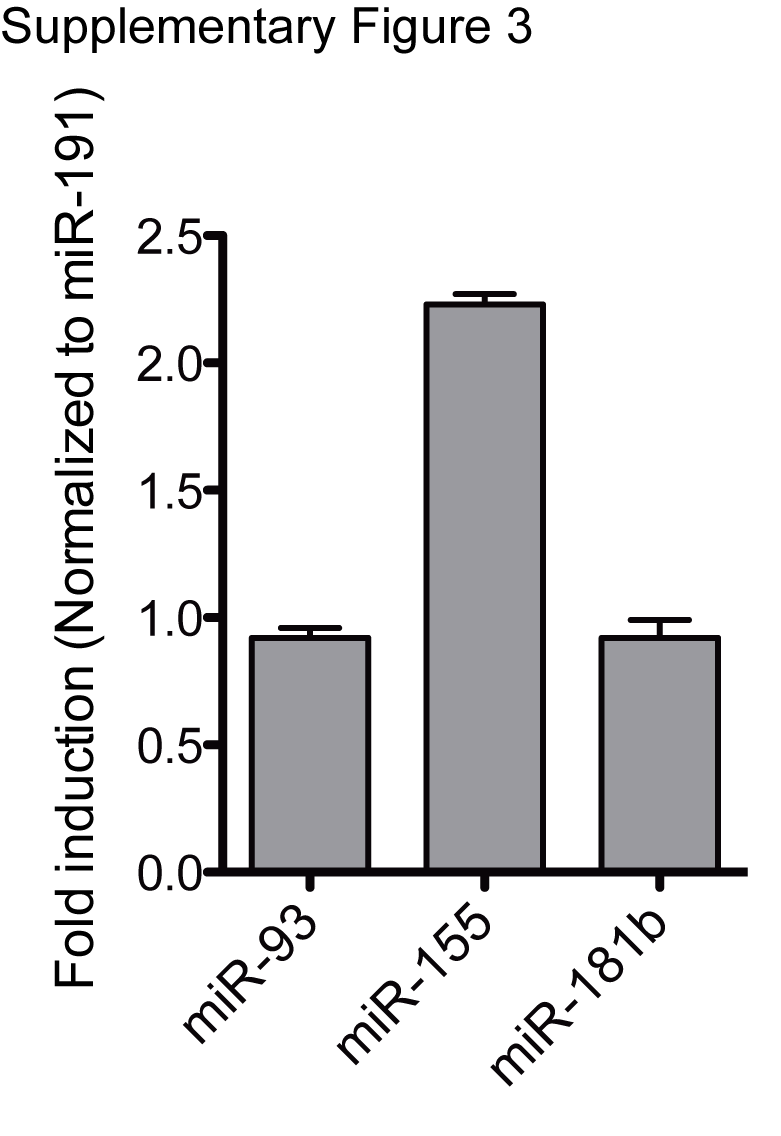

Supplement: Figure S3 — KSHV infection does not dramatically upregulate expression of endogenous miRNA regulating AID. Primary tonsillar cells were infected with KSHV by co-culture with reactivated iSLK.219 cells. After day 3 of co-culture infected, GFP+ and uninfected, GFP− B cells were sorted and total RNA harvested. Relative expression of miR-93, miR-155 and miR-181b was assessed via qRT-PCR analysis. Presented is fold induction of miRNA in infected relative to uninfected cells. Data are normalized to the expression of miR-191. Error bars (SD) are derived from triplicates. Shown is one representative experiment out of three performed. (TIF) [file ppat.1003748.s003.tif]
