# Supplementary material for: A Role for Host Activation-Induced Cytidine Deaminase in Innate Immune Defense against KSHV
Source: PLoS Pathog. 2013 Nov 7;9(11):e1003748. doi: 10.1371/journal.ppat.1003748 (PMC3820765; doi:10.1371/journal.ppat.1003748)
Supplement: Table S1 — Sequences of DNA oligos used in experimental procedures. The table contains DNA sequences for primers and probes used for each indicated gene. The application is specified in column two. When applicable Fwd refers to the forward primer, Rev refers to the reverse primer. (DOCX) [file ppat.1003748.s004.docx]

Table S1. Sequences of DNA oligos used in experimental procedures.

| **Target** | **Application** | **Sequence** |
| --- | --- | --- |
| AID | qPCR primers | Fwd: TTC GCA ATA AGA ACG GCT GCC  Rev: TCG CAG AAA GTC GGC CAC AT |
| MICA | qPCR primers | Fwd: ATG TCC TGC CTG ATG GGA ATG GAA  Rev: CAG CAG CAA CAG CAG AAA CAT GGA |
| MICB | qPCR primers | Fwd: TGG ATC TGT GCA GTC AGG GTT TCT  Rev: TGA GGT CTT GCC CAT TCT CTG TCA |
| ULBP2 | qPCR primers | Fwd: AAC TGC GTG ACA TTC AGC TGG AGA  Rev: TCC AGG ATG AAC CGT TGT CCA CAT |
| ULBP3 | qPCR primers | Fwd: AGT TCA GCT TCG ATG GAC GGA AGT  Rev: AGC CAG CTC CTT GCA GTC TCT CAT T |
| GAPDH | qPCR primers | Fwd: GAA ATC CCA TCA CCA TCT TCC AGG  Rev: GAG CCC CAG CCT TCT CCA TG |
| LANA | qPCR primers | Fwd: TGC TTC TTC TGC AAT CTC CGC  Rev: TGA CTT CGC CAA CCG TAG TGT |
| RTA | qPCR primers | Fwd: TAA TGT CAG CGT CCA CTC CTG  Rev: GGT AAC CTG CAA CAA CGT AAC |
| K1 | qPCR primers | Fwd: GCGCGTTGTGCCAATATAACT  Rev: ACCACACATGGTTCCTATCAG |
| K8.1 | qPCR primers | Fwd: AAG AGG GTT GGA GTG GAC AGG  Rev: ACC CAG AGG CAG ACG TAT CTT |
| LANA gDNA | qPCR primers | Fwd: TGCTTCTTCTGCAATCTCCGC  Rev: TGACTTCGCCAACCGTAGTGT |
| AID gDNA | qPCR primers | Fwd: ATGTGGCCGACTTTCTGCGA  Rev: AGGTCATGATGGCTATTTGCACCC |
| K12-5 Mut 950 | AID 3’UTR mutagenesis | Fwd:cctgggaTGCACGATaagtgtcaacgtttttctatgacttttaggtagga  Rev: gacacttATCGTGCAtcccaggtcctgcagttctgttattc |
| K12-5 Mut 1051 | AID 3’UTR mutagenesis | Fwd: tatatcaACCAGGAAtattatttgattcatttgagttaacagtggtgtta  gtgatagat  Rev: aataataTTCCTGGTtgatataaaaacatttgatcctctcaccatgcttt  ttagg |
| K12-11 Mut | AID 3’UTR mutagenesis | Fwd: ctccaaCTCCAACCtatccaatcatgcgctgtatgttttaatcagcaga  Rev:ttggataGGTTGGAGttggagagatggtttggggtcacaatcacccaga |
| miR-K12-5 | Northern Blot Probe | ACC GGC AAG TTC CAG GCA TCC TA |
| miR-K12-11 | Northern Blot Probe | TCG GAC ACA GGC TAA GCA TTA A |
| Luciferase | Northern Blot Probe | ACC GCC TGA AGT CTC TGA TTA A |
| U6 | Northern Blot Probe | GAA TTT GCG TGT CAT CCT TGC GCA GGG GCC ATG CTA A |
| Neg Ctr  shRNA | Cloning | CCTAAGGTTAAGTCGCCCTCGCTCGAGCGAGGGCGACTTAACCTTAGG |
| Anti-AID  shRNA | Cloning | GCCATCATGACCTTCAAAGATCTCGAGATCTTTGAAGGTCATGATGGCTTTTTG |
| Universal probe | miRNA qPCR | Rev: GAA CAT GTC TGC GTA TCT CAG A |
| miR-191 | miRNA qPCR | Fwd: CAA CGG AAT CCC AAA AGC AG |
| miR-155 | miRNA qPCR | Fwd: TTA ATG CTA ATC GTG ATA GGG GT |
| miR-181b | miRNA qPCR | Fwd: AAC ATT CAT TGC TGT CGG TGG |
| miR-93 | miRNA qPCR | Fwd: CAA AGT GCT GTT CGT GCA G |
