# Supplementary material for: A Role for Host Activation-Induced Cytidine Deaminase in Innate Immune Defense against KSHV
Source: PLoS Pathog. 2013 Nov 7;9(11):e1003748. doi: 10.1371/journal.ppat.1003748 (PMC3820765; doi:10.1371/journal.ppat.1003748)
Supplement: Text S1 — Supporting materials and methods. (DOCX) [file ppat.1003748.s005.docx]

**Supporting Materials and Methods**

**Cell proliferation assays**

BCBL-1 stable transductants were counted and plated at 4x10^5^ cells/ml in individual wells of a 12-well plate in replicates. 24hrs later cells in each well were re-counted and doubling time determined. Additionally, BCBL-1 stable transductants were lab**e**led with Cell Proliferation Dye eFluor 670 (eBioscience) per manufacturer’s instruction. At indicated time points, dilution of the dye, and hence, proliferation was assessed by flow cytometry.

**Quantification of miRNA expression by qRT-PCR**

Total RNA was reverse-transcribed using cDNA Synthesis kit for miRNA (OriGene). miRNA expression was assessed by quantitative RT-PCR using miRNA-specific forward and universal reverse primers.
